# Integrating EEG and Ensemble Learning for Accurate Grading and Quantification of Generalized Anxiety Disorder: A Novel Diagnostic Approach

**DOI:** 10.3390/diagnostics14111122

**Published:** 2024-05-28

**Authors:** Xiaodong Luo, Bin Zhou, Jiaqi Fang, Yassine Cherif-Riahi, Gang Li, Xueqian Shen

**Affiliations:** 1 The Second Hospital of Jinhua, Jinhua 321016, China; lxdjhey@163.com; 2 College of Mathematical Medicine, Zhejiang Normal University, Jinhua 321004, China; binzhou2023@zjnu.edu.cn; 3 College of Engineering, Zhejiang Normal University, Jinhua 321004, China; fjq0971@zjnu.edu.cn; 4 College of Computer Science and Technology, Zhejiang Normal University, Jinhua 321004, China; yassiin.riahii@gmail.com

**Keywords:** generalized anxiety disorder (GAD), electroencephalogram (EEG), functional connectivity, feature selection, machine learning

## Abstract

Current assessments for generalized anxiety disorder (GAD) are often subjective and do not rely on a standardized measure to evaluate the GAD across its severity levels. The lack of objective and multi-level quantitative diagnostic criteria poses as a significant challenge for individualized treatment strategies. To address this need, this study aims to establish a GAD grading and quantification diagnostic model by integrating an electroencephalogram (EEG) and ensemble learning. In this context, a total of 39 normal subjects and 80 GAD patients were recruited and divided into four groups: normal control, mild GAD, moderate GAD, and severe GAD. Ten minutes resting state EEG data were collected for every subject. Functional connectivity features were extracted from each EEG segment with different time windows. Then, ensemble learning was employed for GAD classification studies and brain mechanism analysis. Hence, the results showed that the Catboost model with a 10 s time window achieved an impressive 98.1% accuracy for four-level classification. Particularly, it was found that those functional connections situated between the frontal and temporal lobes were significantly more abundant than in other regions, with the beta rhythm being the most prominent. The analysis framework and findings of this study provide substantial evidence for the applications of artificial intelligence in the clinical diagnosis of GAD.

## 1. Introduction

### 1.1. Background

Generalized anxiety disorder (GAD) is a common anxiety disorder characterized by excessive and persistent worry, tension, and anxiety that extends to multiple aspects rather than being limited to specific events or situations [1]. GAD can negatively impact quality of life, family relationships, financial responsibilities, cognitive functioning, and physical health. Statically, GAD is the most prevalent anxiety disorder, and its prevalence has been increasing annually with the development of society. In China, the prevalence rate of GAD reaches around as high as 5.17% and its lifetime prevalence rate is about 4.66% [2]. It marks a percentage of 3.1% in the annual prevalence rate of United States and 6% of the incidence rate in the United Kingdom [3,4]. Despite the increasing attention to GAD in recent years, the rate of patient seeking treatment and clinical recognition remains low, significantly hindering timely diagnosis and treatment for individuals with GAD. 

The etiology of GAD typically involves multiple factors. Firstly, the family history and genetic factors may increase an individual’s susceptibility to the disorder [5,6]. Secondly, stress related to life events and the surrounding environment is also an imperative factor in triggering it, particularly, work-related stress, interpersonal relationship issues, health concerns, etc. [7]. Furthermore, individual personality traits, such as excessive caution and sensitivity, may also increase the likelihood of developing the disorder [8]. Neurobiological factors, such as imbalances in brain chemicals, particularly neurotransmitters involved in emotional regulation, are also identified to be associated with GAD [9]. Therefore, considering the complex influences of genetics, life events, individual characteristics, and neurobiological factors [10], it is difficult to have an in-depth and thorough understanding of the etiology of GAD and make an accurate diagnosis of GAD. 

Currently, GAD diagnosis typically relies on the evaluation by a psychiatrist based on the Diagnostic and Statistical Manual of Mental Disorders (DSM-5) and a thorough assessment such as the Hamilton Anxiety Rating Scale (HAMA). It is important to note that HAMA cannot be used as a definitive diagnostic tool for anxiety disorders; it is primarily employed to assess the severity of symptoms and heavily relies on subjective responses from patients, which may introduce subjective evaluation biases [11,12]. GAD patients are usually divided into three categories based on the HAMA scores: mild anxiety, moderate anxiety, and severe anxiety [13]. This three-level classification scheme helps medical professionals better understand the symptoms of patients, develop personalized treatment plans, and provide appropriate support and assistance to patients. In actual treatments, due to the diverse manifestations of anxiety disorders, unpredictable courses, and variable treatment responses, their detection, diagnosis, and management often pose challenges to clinicians. When grading and assessing anxiety disorders, patients’ sensory perceptions and expressions vary, making it difficult to objectively and intuitively assess, inevitably leading to the problem of inaccurate grading assessments [14].

However, an electroencephalogram (EEG) can record the electrical activity of the brain’s cortical neurons and synapses. It is a neuroimaging technique that objectively reflects the functional state of the brain [15]. Compared to other physiological signals such as magnetoencephalography (MEG), functional near-infrared spectroscopy (fNIRS), and functional magnetic resonance imaging (fMRI), EEG contains rich information about brain activity due to its real-time insight into brain dynamics, aiding in understanding neural functioning, identifying abnormalities, and diagnosing disorders. Moreover, cognitive neuroscience and its millisecond-level high temporal resolution made studying the real-time patterns of brain activity possible and it has been successfully applied in the clinical assessment of mental health conditions [16]. The EEG technique has potential in detecting different stress levels, depression, and various brain diseases [17]. Currently, EEG is widely used in the diagnosis of GAD. It has been proved that GAD is highly associated with EEG rhythms, especially high-frequency bands [18,19], and the beta rhythm in GAD groups has significantly changes in the power, complexity, and functional connectivity based on EEG signals [3]. Meanwhile, the brain functional network structure deteriorates with the increase in generalized anxiety disorder (GAD) severity based on functional connectivity analysis [20]. We can make the bold assumption that EEG can provide an efficient approach for the objective quantification of GAD.

### 1.2. Related Works

Machine learning combined with EEGs has used in a series of studies in the field of anxiety disorder diagnosis [21,22]. Machine learning can provide new methods for decoding and characterizing EEG information from GAD patients. Nowadays, the EEG-assisted diagnosis of GAD is categorized into three main scenarios: binary classification (normal control and GAD), triple classification (normal control, low-level GAD and high-level GAD), and quadruple classification (normal control, mild GAD, moderate GAD, and severe GAD). It is easy to deduce that the dichotomy method is by far the most popular and more accurate both in machine learning and deep learning. For example, Shen et al. introduced a multidimensional EEG signal feature analysis framework and integrated machine learning methods, achieving a classification accuracy as high as 97.83% [3]. Liu et al. proposed a deep learning model of a multi-scale spatial–temporal local sequential and global parallel convolutional model for normal control and GAD classification and achieved an accuracy rate of 99.47% high-frequency EEG data ranging from 10 to 30 Hz [23]. However, there are fewer studies related to triple GAD classification. Only Qi et al. have reported the difference analysis in brain functional network structures between high-level GAD and low-level GAD and found that the brain functional network structure deteriorates with the increase in GAD severity [24]. But, no machine learning classification studies have been conducted on this triple classification.

Currently, existing studies have employed different classification models on quadruple GAD classifications. For example, Al-Ezzi et al. used fuzzy entropy and machine learning algorithms for four-level GAD and obtained an accuracy of 86.93% [25] and utilized deep learning models and gained an accuracy of up to 92.86% [26]; Mohammad et al. achieved an accuracy of 94.90% and 92.74% in a binary and four-level classification of anxiety disorders using the random forest classification model based on θ and β frequency bands [27]; Li et al. employed a support vector machine classification model for four anxiety states classification, achieving a maximum accuracy of 62.56% [28]. However, the accuracy of these methods is low and cannot meet the needs of the graded diagnosis of disease severity, failing to meet the more detailed requirements of clinical disease diagnosis [29]. In actual clinical practice, due to the complexity of anxiety stages, simply diagnosing the presence or absence of GAD is no longer sufficient to meet the identification needs of early and severe anxiety. The use of EEG technology combined with artificial intelligence algorithms may improve the four-level classification accuracy of GAD, thus enhancing diagnostic efficiency. However, there are limited reports on the identification rates for four-level GAD, and the reported studies still needs for algorithmic enhancement and analysis of brain function mechanisms.

Here, this study attempts to propose an algorithm framework to achieve the best classification accuracy for GAD four-level classification, combining feature selection algorithms to obtain the optimal combination of EEG features when performance is at its best and studying the differences in brain function among GAD patients of different severity based on the optimal feature subset. More specifically, in the Introduction section, the incidence of GAD and the current status of diagnosis are introduced to initiate this research on combining EEGs and ensemble learning to carry out multilevel GAD diagnosis. In the Materials and Methods section, the subject information of this study, the EEG data collection, preprocessing, and analysis processes, as well as the artificial intelligence algorithms and model evaluation metrics are described in detail. In the Results section, the classification results of the four-level GAD classification for different time windows, sample enhancement, and feature selection are presented, and the optimal set of features is analyzed in detail. In the Discussion section, the effects of time windows, functional connectivity, and beta rhythm on the four-level GAD classification are discussed, and the limitations of this study are given. In the Conclusion section, the main findings of this study and future research directions are summarized.

## 2. Materials and Methods

### 2.1. Participants

**Figure 1:**
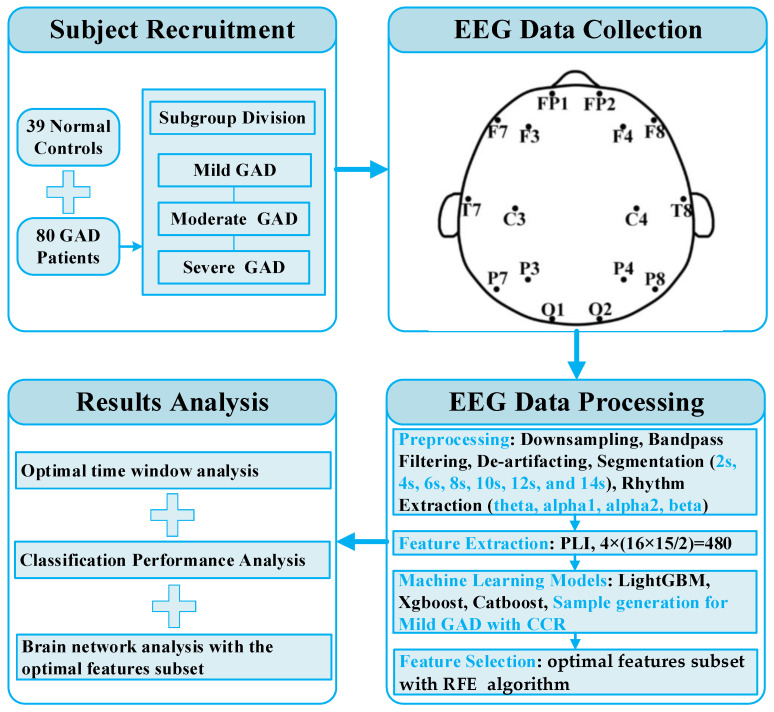
Analysis framework for this study.

GAD patients were recruited from local hospitals, including 39 normal controls and 80 GAD patients, totaling 119 participants, with an age range of 22 to 68 years. All recruited subjects were diagnosed by psychiatric specialists based on the criteria for GAD outlined in the fifth edition of “The Diagnostic and Statistical Manual of Mental Disorders” (DSM-5). These participants also completed the Hamilton Anxiety Rating Scale (HAMA). The overall analytical framework of this study is shown in Figure 1. In this study, specific conditions were imposed on each participant to ensure the accuracy and reliability of the data: (1) all participants had to be right-handed; (2) participants could not have other psychiatric disorders such as epilepsy, neurodegenerative diseases, stroke, or schizophrenia; (3) participants could not have severe physical disorders; (4) participants could not have a history of drug or alcohol abuse; (5) participants could not have any record of brain injury; and (6) participants had to ensure adequate sleep the night before data collection and avoid smoking, drinking coffee, and strong tea within 2 h before the experiment. Additionally, based on the literature [11] and the total score of the Hamilton Anxiety Rating Scale (HAMA), participants were categorized into different severity levels, including normal, mild, moderate, and severe, as shown in Table 1. 

### 2.2. EEG Data Recording and Preprocessing

The EEG data collection was conducted in a professional EEG room, with each participant required to provide 10 min of resting-state EEG data. Participants were instructed to keep their eyes closed, remain awake, and relax during the data collection process. The Nicolet EEG TS215605 was used for EEG recordings, and the electrodes were placed according to the international 10–20 system. A total of 16 electrodes were selected, specifically FP1, FP2, F3, F4, C3, C4, P3, P4, O1, O2, F7, F8, T3, T4, T5, and T6. Additionally, the left and right mastoids were used as reference electrodes. During data collection, the sampling frequency was set at 250 Hz, and electrode impedance was required to be less than 5 kΩ. The collected EEG data underwent the following preprocessing steps: (1) EEG data were down sampled to 125 Hz and filtered using a fourth-order Butterworth bandpass filter between 4 and 30 Hz. (2) Fast independent component analysis (ICA) was utilized to remove artifacts from the EEG data, including eye blink artifacts, electrocardiogram artifacts, and electromyogram artifacts. (3) The continuous EEG data were segmented into 2 s, 4 s, 6 s, 8 s, 10 s, 12 s, and 14 s segments according to the pre-experimental classification findings and insights from the existing literatures, and the sample sizes were calculated for four categories: normal controls, mild GAD, moderate GAD, and severe GAD, as shown in Table 2. (4) Different rhythmic EEG signals, including theta waves (4–8 Hz), alpha1 waves (8–10 Hz), alpha2 waves (10–13 Hz), and beta waves (13–30 Hz), were extracted from each segment of the EEG data. Through these steps, the collected EEG data underwent a series of meticulous preprocessing procedures for subsequent analysis and research.

### 2.3. Feature Extraction

In this study, the Phase Lag Index (PLI) was extracted for the identification of four levels of anxiety disorder patients. The PLI is a measure of relative phase distribution asymmetry derived from the instantaneous phase of a pair of time series (in this case, electroencephalogram signals from a pair of electrodes). The PLI can effectively reduce the effects of volumetric conduction and noise, thus reflecting more accurately the synchronization and signal transmission between different brain regions. For a pair of bandpass-filtered time series, $x_{1}\left(t\right)$ and $x_{2}(t)$, and the instantaneous phase calculated using the Hilbert transform [30], the computation of the PLI is described by Equation (1).

(1) $$z_{i}\left(t\right)=x_{i}\left(t\right)+j\frac{1}{\pi }P.V.\int_{-\infty }^{\infty }\frac{x_{i}\left(t\right)}{t-\tau }d\tau$$

In this equation, *P.V.* represents the Cauchy principal value. The calculation formula for the PLI of two lead electroencephalogram signals is shown in Equation (2).

(2) $$PLI=\left|\left\langle \mathrm{sign}\Delta \varphi \left(t\right)\right\rangle \right|=\left|\left\langle \mathrm{sign}\left(\arg\left(\frac{z_{1}\left(t\right)z_{2}^{*}\left(t\right)}{\left|z_{1}\left(t\right)\right|\left|z_{2}\left(t\right)\right|}\right)\right)\right\rangle \right|$$

In this equation, |•| denotes taking the mean, <•> represents taking the absolute value, and Δ*φ*(*t*) represents the phase difference between the two signals. The value of the PLI ranges from 0 to 1, indicating the degree of phase synchronization between brain regions. A PLI value close to 0 means no phase synchronization, while a value close to 1 indicates complete phase synchronization.

In this analysis, four specific frequency bands were extracted. For each frequency band, the PLI was calculated for all pairwise combinations of leads. As a result, N × (N − 1)/2 features were obtained, where N represents the number of leads (N = 16 in this study). Therefore, a total of 4 × 120 = 480 features were computed in this experiment.

### 2.4. Machine Learning Models Introduction

#### 2.4.1. Models

The ensemble learning models based on decision trees have shown excellent classification performance and have been widely applied in disease detection. In this experiment, LightGBM (Light Gradient-Boosting Machine) [31], Xgboost (extreme gradient boosting) [32], and Catboost (categorical boosting) [33] were employed for the classification of four levels of anxiety disorder patients. The learning models are as follows: 

(1) LightGBM is an efficient machine learning algorithm based on a gradient-boosting decision tree (GBDT), optimized for large-scale datasets and high-dimensional features. It utilizes a technique called “Histogram-based Decision Tree” to discretize feature values and construct histograms, accelerating the training process while reducing memory consumption and computational complexity. In each iteration, it uses the gradient boosting algorithm to build decision trees and improves model performance by minimizing the loss function. Compared to traditional GBDT, it also introduces an optimal split algorithm based on leaf nodes, which enhances model accuracy by selecting the best splitting points accurately. Additionally, LightGBM supports parallel training and prediction, offers a range of tuning options, and provides methods for feature importance evaluation.

(2) Xgboost is a machine learning algorithm based on a GBDT widely applied in the fields of machine learning and data mining. It uses decision trees as base learners and constructs a powerful ensemble learning model by iteratively optimizing the gradient of the loss function. The principle can be summarized as follows: during the construction of each tree, the gradient boosting algorithm is used to minimize the loss function, and regularization terms are introduced to control the model complexity and prevent overfitting. Moreover, XGBoost adopts a custom optimization algorithm called “approximate greedy algorithm” that efficiently utilizes the second-order gradient information of features to accelerate the training process. Finally, the predictions of each weak learner are aggregated to obtain the final prediction output.

(3) CatBoost is the third GBDT-based algorithm, following XGBoost and LightGBM, specifically designed to handle categorical features in classification or regression tasks. CatBoost can automatically handle categorical features without the need for one-hot encoding or label encoding, simplifying the data preprocessing steps and reducing the risk of overfitting. During the training process, this model utilizes a weighted symmetric tree-based algorithm for second-order gradient boosting and state-of-the-art optimization techniques, achieving outstanding performance and the efficient handling of large-scale datasets. It possesses the ability for automatic hyperparameter tuning, automatically adjusting parameters such as the learning rate and tree depth, reducing the complexity of hyperparameter tuning, and making model training more convenient. It exhibits strong robustness when dealing with missing values, outliers, and noisy data, enabling it to handle complex real-world situations and improve model generalization. It provides an intuitive model interpretation feature, allowing for understanding the contribution of features to predictions, making the model results more interpretable and understandable. Overall, CatBoost is a powerful tool for solving classification and regression problems, characterized by its excellent categorical feature-handling capability, high performance, automatic hyperparameter tuning, and robustness.

#### 2.4.2. Parameters Optimization

Optimizing hyperparameters plays a crucial role in the performance and generalization ability of tree-based machine learning models, as these models often have a large number of hyperparameters. By optimizing them, the risk of overfitting or underfitting can be reduced, leading to the improved predictive ability and robustness of the model [34]. Therefore, in this study, the Tree of Parzen Estimators (TPEs) algorithm was used to search for the optimal combination of hyperparameters and enhance model performance. Compared to traditional grid search or random search methods, the TPE algorithm is an efficient hyperparameter optimization approach based on the hyperopt Python package. It effectively utilizes historical data, accelerates the search process, and improves optimization efficiency. As a result, it has lower time complexity and can find a relatively optimal combination of hyperparameters within a relatively short period.

The algorithm mainly encompasses three key steps to ensure the effectiveness and feasibility of the optimization process. Firstly, defining the optimization objective function serves as the foundation of the TPE algorithm. This study used the f1_macro score, which measures the model’s prediction performance compared to the ground truth values on the test set, as the optimization objective. Secondly, defining the search space for hyperparameters is crucial as it was used by the model presented in Table 3, Table 4 and Table 5 in this study, whereas the selection and range of machine learning optimization parameters were referenced from the autogluon automatic machine learning framework [35]. Lastly, determining the number of hyperparameter search iterations is important. To control the iteration, count of the search process, the maximum number of search iterations in this study was set to 30. By combining these three steps, the TPE algorithm can rapidly and effectively explore the hyperparameter space, thereby enhancing the performance and generalization ability of the machine learning model.

#### 2.4.3. Evaluation Metrics

**Figure 2:**
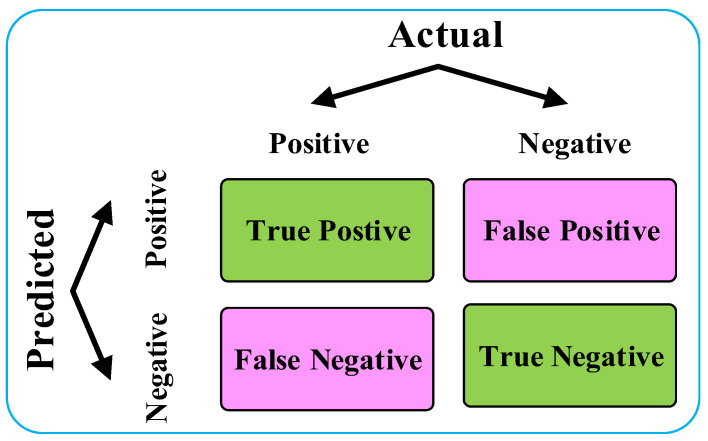
Confusion matrix.

The confusion matrix is commonly used to evaluate the performance of binary classification models, as shown in Figure 2. In this matrix, the important metrics are defined as follow: true positives (TPs) represents the number of samples correctly predicted as positive; false positives (FPs) indicates the number of samples incorrectly predicted as positive when they are actually negative; false negatives (FNs) means the number of samples incorrectly predicted as negative when they are actually positive; and true negatives (TNs) represents the number of samples correctly predicted as negative. By utilizing the confusion matrix, multiple performance metrics can be derived to assess the model’s performance, thus providing robust support for model design and optimization.

Common evaluation metrics for binary classification models based on the confusion matrix include accuracy, precision recall, and F1 score, as shown in Equations (3)–(6):

(3) $$Accuracy=\frac{TP+TN}{TP+TN+FP+FN}$$

(4) $$Precision=\frac{TP}{TP+FP}$$

(5) $$Recall=\frac{TP}{TP+FN}\;$$

(6) $$F1=\frac{TP+TN}{2TP+FP+FN}\;$$

Due to the multi-class nature of the task addressed in this study, as well as the presence of class imbalance, a wide-ranging evaluation of model performance was conducted by considering multiple evaluation metrics such as accuracy, F1_macro, Gmean_macro, and kappa. These metrics, all ranging from 0 to 1, are used to accurately measure the model’s performance level, with values closer to 1 indicating better performance. The following paragraph provides a detailed introduction to the F1_macro, Gmean_macro, and kappa metrics:

(1) F1-Macro calculates the F1 score for each class and then computes the arithmetic mean of these F1 scores, as shown in Equation (7).

(7) $$F1\text{-}\mathrm{Macro}=\frac{{F1}_{1}+{F1}_{2}+\ldots +{F1}_{C}}{C}$$

*C* represents the number of classes, and ${F1}_{1}$, ${F1}_{2}$, ..., ${F1}_{c}$ represent the F1 scores for each class.

(2) Gmean-macro assigns equal weight to the performance of all classes, and the calculation formula is shown in Equation (8).

(8) $$\mathrm{Gmean}\text{-}\mathrm{macro}=\frac{\sqrt{{Recall}_{1}\times {Recall}_{2}\times \ldots \times {Recall}_{C}}}{C}$$

(3) Kappa is a metric used to assess the consistency between a classifier and a random one, suitable for multi-class classification problems, with the calculation formulas shown in Equations (9) and (10).

(9) $$\kappa =\frac{P_{o}-P_{e}}{1-P_{e}}$$

where $P_{o}$ refers to the observed classification consistency, i.e., the accuracy of the test. $P_{e}\;represents$ the expected classification consistency, which is obtained by calculating the expected accuracy for each category and averaging them. $P_{o}\;and\;P_{e}$ in the formula can be computed based on the confusion matrix, as detailed below:

(10) $$P_{e}=\frac{\left(TP+FP)\times (TP+FN)+(FN+TN)\times (FP+TN\right)}{(TP+TN+FP+FN{)}^{2}}$$

The present study employed repeated 5-fold cross-validation to reduce bias in the classification results, with four sets of data used for model training and one set for testing. All models underwent three repetitions of 5-fold cross-validation, and the final result was obtained by averaging the results of all test sets.

#### 2.4.4. Data Generation Algorithm

Due to the presence of class imbalance in our study data, with fewer samples in the mild GAD category compared to other classes, the CCR (combined cleaning and resampling) algorithm [36] was employed to improve model performance by resampling the minority class. CCR is a cleaning and resampling algorithm used for imbalanced data classification problems, consisting of two steps: firstly, it removes majority class samples that are too close to the minority class to clean the decision boundary; then, it selectively oversamples the minority class samples to generate a large number of synthetic samples around unsafe samples, aiming to reduce bias towards the minority class.

### 2.5. Feature Selection

In this study, a total of 480 features were extracted, resulting in a high-dimensional feature space. Therefore, feature selection algorithms were applied to remove irrelevant and redundant features, selecting the most relevant and informative ones to achieve dimensionality reduction and improve model interpretability. In this research, the RFE (Recursive Feature Elimination) algorithm was utilized for feature selection as it progressively eliminates features with lower contributions to the predictive performance, thereby selecting an optimal subset of features. The RFE process implemented in this study is as follows:

(1) The dataset was divided using K-fold cross-validation, where the training set was used to train the classifier and obtain its parameters. Thus, the testing set was then used, and in conjunction with the feature importance method available in Python for tree model ensembles, the importance of each feature was calculated. The importance scores of the features were computed for each fold of the testing set, and the average importance score was determined. The features were then sorted based on their importance scores.

(2) The feature with the lowest score in the current feature set was removed, and the model performance was evaluated.

(3) Steps (1) and (2) were repeated until only one feature remained in the feature set. Here, the concept of “early stopping”, commonly used in deep learning training models, was introduced. If the model performance metric did not improve for a consecutive number of iterations (set as 10 in this study), the iteration process was stopped. Based on the model performance of each feature subset, the latter with the highest accuracy was identified as the optimal one.

## 3. Results

**Figure 3:**
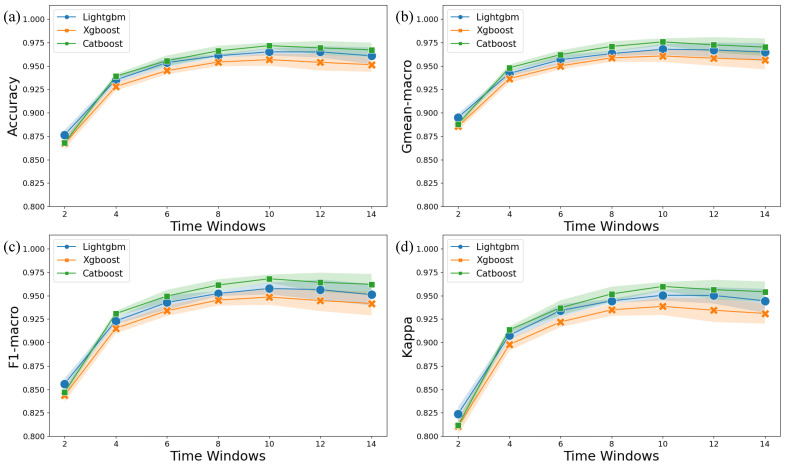
GAD four-level classification performance of accuracy (**a**), Gmean-macro (**b**), F1-macro (**c**), and Kappa (**d**) at various time windows. The shadow represents the standard deviation.

In order to identify the appropriate time window for EEG data to achieve optimal classification performance, this study evaluated the classification performance of anxiety disorder using accuracy, Gmean-macro, F1-macro, and Kappa across seven time windows (2 s, 4 s, 6 s, 8 s, 10 s, 12 s, and 14 s) as shown in Figure 3. As the length of the time window increased, the accuracy, recall rate, F1 score, and Kappa index of the Lightgbm, Xgboost, and Catboost classification models gradually improved before stabilizing. All models and metrics performed optimally in the 10 s time window. Compared to the 2 s time window, the accuracy of the GAD four-level classification in the 10 s time window increased by approximately 10%. Additionally, the trend chart indicates low model variability and high stability across all models.

Upon comparing the GAD four-level classification performance of each model within 10 s time windows, as shown in Table 6, the results indicate that the Catboost model exhibits the best performance across all metrics, specifically, accuracy 97.2 ± 0.3%, F1-macro 96.8 ± 0.5%, Gmean-macro 96.0 ± 0.5%, and Kappa 97.6 ± 0.4%. As depicted on the right side of Table 6, the recognition accuracy of each model in the normal, mild anxiety, moderate anxiety, and severe anxiety groups is presented. The results demonstrate that the Catboost model achieves the highest accuracy, with accuracy rates of 97.6 ± 0.4% for the normal group, 92.4 ± 2.8% for the mild anxiety group, 98.5 ± 0.3% for the moderate anxiety group, and 96.4 ± 0.8% for the severe anxiety group. Due to the relatively smaller sample size in the mild anxiety group compared to the other groups, the data exhibit class imbalance, leading to lower accuracy in this group.

**Figure 4:**
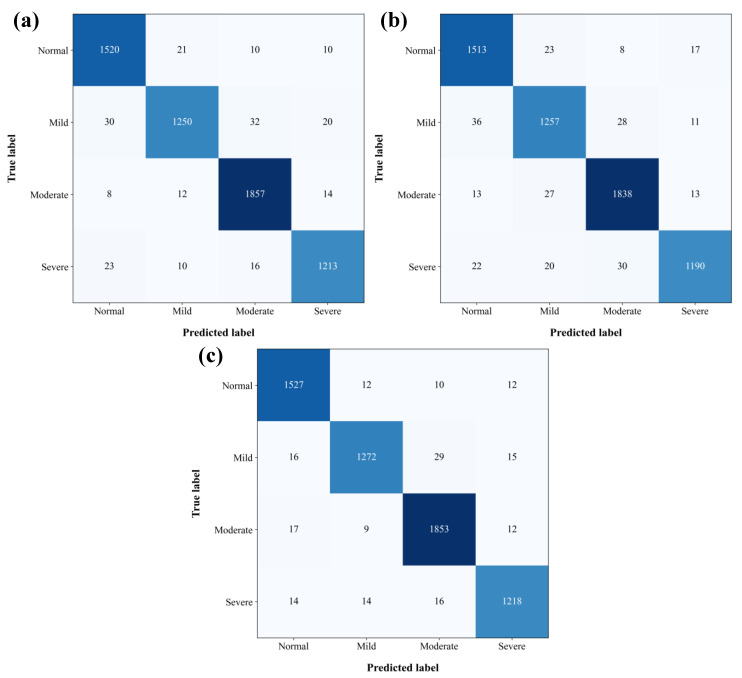
Confusion matrices for LightGBM (**a**), Xgboost (**b**), and Catboost (**c**) models according to Table 7. The darker color of the square indicates a larger value.

Due to the smaller sample size of the mild anxiety group compared to other categories, the CCR algorithm was used in this study to perform the resampling of the minority class. Particularly, for 10 s time windows, the training sample size of the mild anxiety group was increased from 356 to 1424. As shown in Table 7 and Figure 4, the overall performance of each model improved by varying degrees after generating CCR data, particularly in the mild anxiety group, where the recognition accuracy significantly increased. Within the mild anxiety group, the accuracy of the Lightgbm model increased from 87.8 ± 4.2% to 93.9 ± 2.8%, the Xgboost model’s accuracy increased from 85.8 ± 3.3% to 94.4 ± 2.8%, and the Catboost model’s accuracy increased from 92.4 ± 2.8% to 95.5 ± 1.4%.

**Figure 5:**
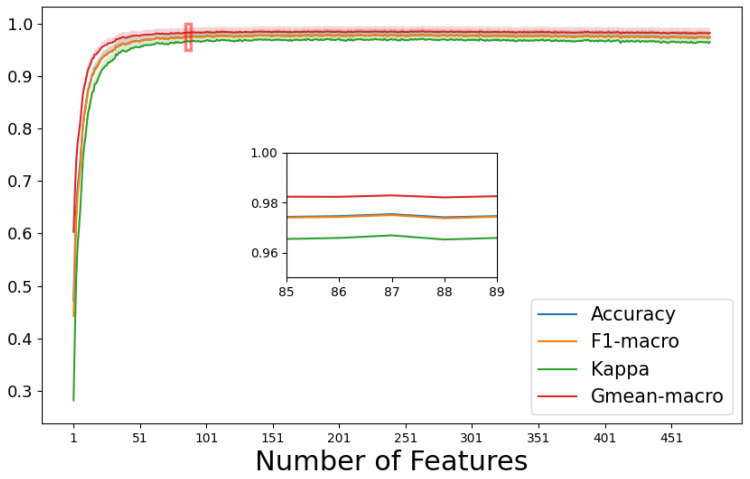
The variation in model metrics along with the iterations of the ranked features (the subplot represents the performance of the optimal feature subset). Accuracy and F1-macro are nearly overlapping; only three lines are visible.

This study applied RFE for feature selection and introduced the “early stopping” concept in deep learning to select the optimal feature subset. As depicted in Figure 5, it shows the variation in performance metrics in the Catboost model during the iterative process, and the subplot represents the performance of the selected optimal subset. The results indicate that after removing irrelevant and redundant features from the initial 480 features, an optimal feature subset of 87 features was achieved. The highest accuracy of the optimal feature subset was 98.1 ± 0.6%, with the highest FI score 98.1 ± 0.6%, kappa 97.3 ± 0.7%, and Gmean-macro 98.8 ± 0.4%.

**Figure 6:**
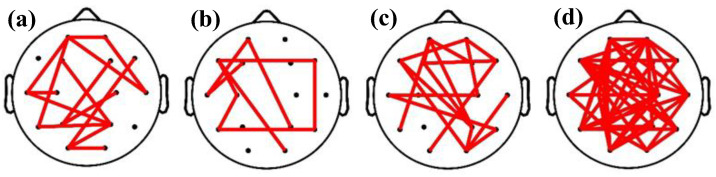
The brain functional connectivity of the optimal feature subset, (**a**) theta, (**b**) alpha1, (**c**) alpha2, (**d**) beta.

**Figure 7:**
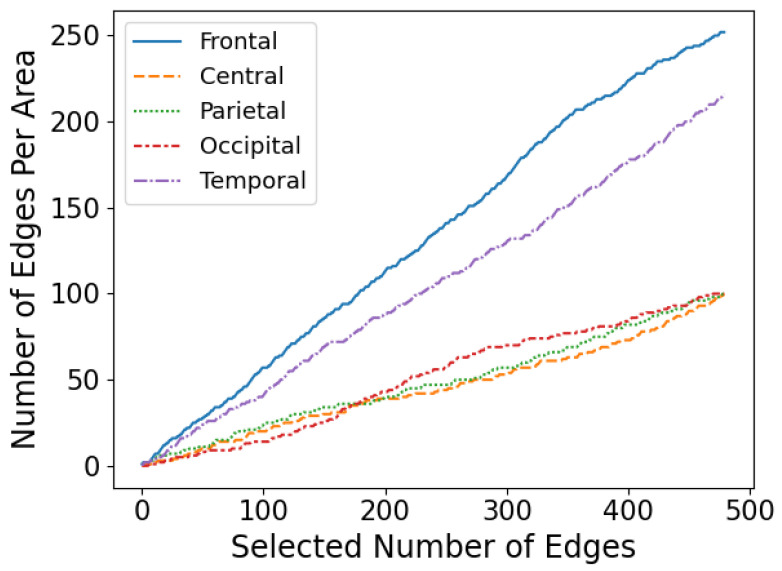
Numbers of connections of each brain region within various feature subsets.

**Figure 8:**
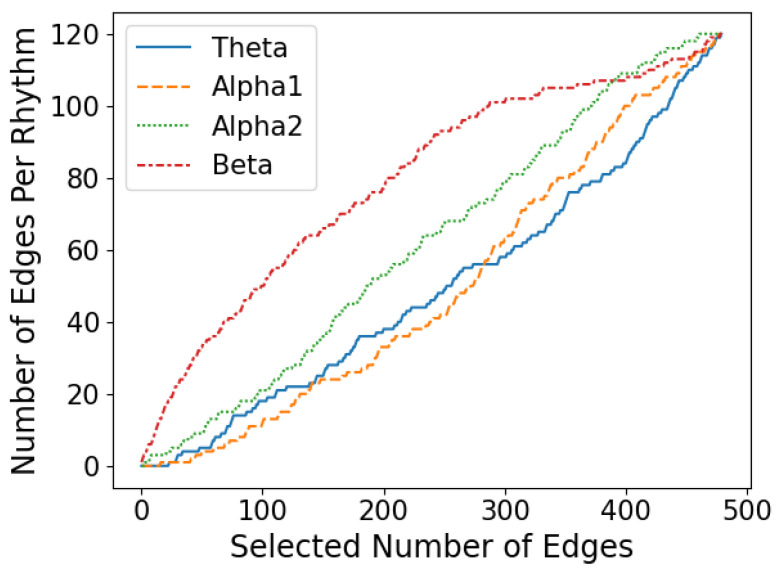
Numbers of rhythms of each brain region within various feature subsets.

Based on the optimal feature subset, the brain functional connectivity network of GAD at four levels is shown in Figure 6, with brain region distribution maps and rhythmic distribution maps shown in Figure 7 and Figure 8, respectively, according to the importance ranking of all features. As depicted in Figure 6, the brain functional connectivity in GAD patients at different levels undergoes reorganization across the entire brain regions. Furthermore, as indicated in Figure 7, this reorganization of the brain network structure is primarily distributed in the frontal and temporal lobes, with no significant differences observed among the central, parietal, and occipital lobes. According to Figure 8, the altered functional connections are primarily distributed in the beta rhythm, with the distribution of the 87 optimal functional connections as follows: θ: 14/87, α1: 9/87, α2: 18/87, and β: 46/87.

## 4. Discussion

In this resting-state EEG study, a machine learning framework was introduced for the four-level classification of healthy individuals, mild GAD, moderate GAD, and severe GAD, and conducted a detailed analysis of the brain functional connectivity at these four levels of GAD. This study revealed that the selection of time windows is crucial for the precision diagnosis of GAD at the four levels, resulting in the 10 s time window achieving the best classification accuracy of 98.1 ± 0.6%. Additionally, the functional connectivity results of the optimal feature subset revealed that GAD functional connections are primarily distributed in the frontal and temporal brain regions, with network characteristics between brain regions predominantly manifesting in the beta rhythm. This further indicates substantial differences in the brain functional network structure among GAD severity levels.

### 4.1. Appropriate Time Window Length Can Achieve Optimal Performance in the Four-Level Diagnosis of GAD

The time window method is widely used for extracting brain functional connectivity, and it is crucial in brain functional connectivity-related research. This study reveals that the appropriate time window length is essential for evaluating the stability and performance of the model. Further analysis indicates that selecting an appropriate time window length can yield the best classification accuracy, while too-short time windows may not fully extract EEG signal features, and too-long time windows may lead to masking variability in features, adversely affecting the performance of the classification model [37]. He et al. analyzed that the length of the time window has a significant impact on the dynamic brain network analysis under different emotional conditions [38]. Nunez et al. also reported that time window length is an important parameter to determine when performing a sliding window analysis, and a 10 s time window was set as the minimum length required to avoid spurious results [39]. Li et al. proposed a technique called brain rhythm sequencing, which found that single-channel data with a time window length of 10 s achieved the highest classification accuracy of 82% in emotion recognition [40]. Azinfar et al. found in their EEG study of epileptic patients that the optimal time window length for feature extraction was 10 s, achieving a detection sensitivity of 83.99% [41]. However, Ouyang et al. have reported that the optimal time window length for extracting EEG emotion signals is 2 s when investigating the effect of different time window lengths on human emotion recognition [42]. In summary, optimizing the selection of appropriate time windows is crucial for different research tasks. This study found that a 10 s time window was optimal for classifying GAD into four levels and positively influenced the recognition accuracy of three models, providing a solid experimental basis for intelligent diagnosis research related to GAD.

### 4.2. Functional Connectivity Features Facilitate the Graded Diagnosis of GAD

In the field of mental disorders, it is necessary to quantify the severity of the disease in order to achieve better diagnostic and treatment outcomes. Functional connectivity can effectively quantify the brain’s functional status and provide an objective assessment of various levels of anxiety symptoms [43]. In the multi-level classification of anxiety disorders, functional connectivity analysis can identify differences in the brain functional networks of patients with different levels of anxiety [20]. The previous research results indicate that functional connectivity features in brain electrical signals often achieve better classification accuracy than other types of features, such as power spectral features and nonlinear dynamical features [19,44]. The existing literature reports that Al-Ezzi et al. gained an accuracy of up to 92.86% for four-level GAD classification [26]. To the best of our knowledge, this is the highest reported four-level classification accuracy known to date. This study, through the selection of time window length and feature selection algorithms, achieved the highest known accuracy of 98.1% using functional connectivity features. In clinical practice, the primary purpose of this grading is to help doctors and clinical experts better understand the patient’s condition and develop appropriate treatment plans. This not only benefits clinical diagnosis and treatment but also facilitates the evaluation of clinical efficacy and improves the clinical cure rate [45].

### 4.3. Functional Connectivity Distributions Significantly Differ across the Four Levels of GAD

The theory and techniques of brain functional networks effectively reveal the functional integration and segregation among different brain regions. In this research, a feature selection algorithm was used to screen out 87 optimal feature subsets from 480 features. The beta frequency band accounted for the highest proportion in this optimal feature subset. It was significantly obvious that the number of connections between the frontal and temporal lobes at beta EEG rhythm was much more significant than other rhythms. Previous researches have reported the significantly increased power of beta rhythm [46,47,48] and a similar distribution of the frontal region [49] for GAD patients. Beta rhythm is commonly associated with states of alertness and arousal in the brain [43,50,51] and mediate higher cognitive functions [52,53]. There is strong evidence from neuroimaging studies suggesting that emotional regulation in GAD patients is associated with abnormalities in the neural circuits of the beta rhythm and frontal lobe margin region [54]. For example, Shen et al. reported that the multidimensional EEG features of beta rhythm have significantly changes in GAD groups across the frontal brain region [3]. Beta rhythm is mainly observed during high cognitive load states, such as thinking, attention, and anxiety [55]. Research by Jang et al. indicated a certain correlation between enhanced beta band activity and symptoms of anxiety disorders [56]. One possible explanation for our findings may be the increased worry/internal cognitive processing of GAD in the form of divergent negatively biased mind wandering during non-specific information processing, leading to the alteration of beta rhythm across the frontal brain region [57]. Qi et al. also demonstrated a significant correlation between functional connectivity and the prefrontal cortex in GAD brain functional connectivity analysis, primarily distributed in the frontal lobe (accounting for approximately 73%) [20]. Wang et al. also reported a similar conclusion that the source signals that play a critical role in GAD classification mostly originate from brain regions such as the prefrontal and temporal lobes [58]. Using precise brain region and rhythm EEG signals for classification can reduce signal clutter, optimize classification features, and achieve higher classification efficiency. Although the conclusions of the above studies are not directly derived from the results of GAD severity grading, these conclusions can support the reliability of this study.

### 4.4. Limitations

Although this study has achieved considerable beneficial results, certain limitations still persist. Firstly, the sample size is limited, and there is an imbalance in the samples. In future research, it is possible to further increase the number of participants to provide an adequate sample for machine learning and balance the number of samples between groups. Secondly, there is a gender imbalance in the mild GAD group, which will be taken into account in future in-depth studies. Thirdly, additional enhancement can be implemented to the existing models to improve classification accuracy and sensitivity in order to optimize the clinical diagnostic capabilities for patients with anxiety disorders. These improvements aim to facilitate more objective and efficient classification and evaluation.

## 5. Conclusions

This study attempts to propose an algorithmic framework for achieving the optimal classification of GAD into four levels. It combines feature selection algorithms to obtain the optimal combination of EEG features for achieving the best classification performance. Furthermore, it investigates the differences in brain functionality among GAD patients with different severity levels based on the optimal feature subset. The results of this research demonstrate that using the Catboost model and a time window of 10 s, a highly accurate classification of the four GAD groups can be achieved with an accuracy rate of 98.1%. Additionally, further exploration of the distribution of brain functional connectivity reveals that the frontal and temporal brain regions exhibit a significantly higher number of functional connectivity compared to the central, parietal, and occipital lobes, with the highest number of connections observed in the β rhythm. These findings are expected to provide important insights for understanding and diagnosing GAD. In future studies, state-of-the-art deep learning techniques and the quantification of GAD severity will positively contribute to the accurate diagnosis and treatment of GAD.

## Figures and Tables

**Table 1:** Demographic and clinical characteristics of the participants.

	Normal	Mild GAD	Moderate GAD	Severe GAD
HAMA score	score < 7	7 ≤ score ≤ 17	18 ≤ score ≤ 24	score > 24
Number	39	9	38	33
Age	38 ± 13	46 ± 15	50 ± 11	49 ± 12
Gender (male/female)	12/27	3/6	10/28	8/25
HAMA mean score	2.3 ± 0.9	13.4 ± 2.8	21.0 ± 2.0	31.9 ± 7.1

**Table 2:** Number of features in different time window lengths.

Time Window Length (s)	Normal	Mild	Moderate	Severe
2	8665	2359	9927	6839
4	4242	1162	4905	3361
6	2676	716	3009	2092
8	2010	561	2389	1608
10	1561	444	1891	1262
12	1325	342	1464	1010
14	896	241	1016	707

**Table 3:** LightGBM optimization variable and range.

Parameter	Description
num_leaves	uniformint [16, 96]
min_data_in_leaf	uniformint [2, 60]
feature_fraction	uniform [0.75, 1]
learning_rate	loguniform [log(5 × 10^−3^), log(0.1)]

**Table 4:** Xgboost optimization variable and range.

Parameter	Description
learning_rate	loguniform [log(5 × 10^−3^), log(0.1)]
depth	uniformint [3, 10]
min_child_weight	uniformint [1, 5]
colsample_bytree	uniform [0.5, 1.0]

**Table 5:** Catboost optimization variable and range.

Parameter	Description
max_depth	uniformint [5, 8]
l2_leaf_reg	uniform [1, 5]
learning_rate	loguniform [log(5 × 10^−3^), log(0.1)]

**Table 6:** The performance of three models at 10-second time windows.

Models	Accuracy	F1-Macro	Gmean-Macro	Kappa	Normal Accuracy	Mild Accuracy	Moderate Accuracy	Severe Accuracy
Lightgbm	96.5 ± 0.4%	95.8 ± 0.4%	95.0 ± 0.6%	96.8 ± 0.6%	97.4 ± 0.4%	87.8 ± 4.2%	97.9 ± 0.5%	96.4 ± 0.3%
Xgboost	95.7 ± 0.7%	94.9 ± 0.8%	93.9 ± 0.9%	96.1 ± 0.6%	96.7 ± 0.7%	85.8 ± 3.3%	97.6 ± 1.1%	95.0 ± 0.5%
Catboost	97.2 ± 0.3%	96.8 ± 0.5%	96.0 ± 0.5%	97.6 ± 0.4%	97.6 ± 0.4%	92.4 ± 2.8%	98.5 ± 0.3%	96.4 ± 0.8%

**Table 7:** After CCR the performance of three models at 10-s time windows.

Models	Accuracy	F1-Macro	Gmean-Macro	Kappa	Normal Accuracy	Mild Accuracy	Moderate Accuracy	Severe Accuracy
Lightgbm	97.1 ± 0.4%	96.1 ± 0.7%	97.4 ± 0.3%	97.1 ± 0.4%	97.4 ± 0.5%	93.9 ± 2.8%	98.2 ± 0.2%	96.1 ± 0.4%
Xgboost	96.8 ± 0.7%	95.7 ± 0.4%	97.0 ± 0.5%	96.8 ± 0.7%	96.9 ± 0.6%	94.4 ± 2.8%	97.2 ± 0.5%	94.3 ± 0.8%
Catboost	95.9 ± 0.7%	94.5 ± 0.5%	96.3 ± 0.7%	95.9 ± 0.7%	97.8 ± 0.4%	95.5 ± 1.4%	98.0 ± 0.4%	96.5 ± 0.5%

## Data Availability

The data presented in this study are available on request from the corresponding author. The data are not publicly available due to protect the privacy of patients.

## References

[1] DeMartini J., Patel G., Fancher T.L. (2019). Generalized Anxiety Disorder. Ann. Intern. Med..

[2] Guo X., Meng Z., Huang G., Fan J., Zhou W., Ling W., Jiang J., Long J., Su L. (2016). Meta-analysis of the prevalence of anxiety disorders in mainland China from 2000 to 2015. Sci. Rep..

[3] Shen Z., Li G., Fang J., Zhong H., Wang J., Sun Y., Shen X. (2022). Aberrated Multidimensional EEG Characteristics in Patients with Generalized Anxiety Disorder: A Machine-Learning Based Analysis Framework. Sensors.

[4] Saramago P., Gega L., Marshall D., Nikolaidis G.F., Jankovic D., Melton H., Dawson S., Churchill R., Bojke L. (2021). Digital Interventions for Generalized Anxiety Disorder (GAD): Systematic Review and Network Meta-Analysis. Front. Psychiatry.

[5] Eugene L., Shih-Jen T. (2020). Gene-Environment Interactions and Role of Epigenetics in Anxiety Disorders. Adv. Exp. Med. Biol..

[6] Stoychev K., Dilkov D., Naghavi E., Kamburova Z. (2021). Genetic Basis of Dual Diagnosis: A Review of Genome-Wide Association Studies (GWAS) Focusing on Patients with Mood or Anxiety Disorders and Co-Occurring Alcohol-Use Disorders. Diagnostics.

[7] Showraki M., Showraki T., Brown K. (2020). Generalized Anxiety Disorder: Revisited. Psychiatr. Q..

[8] Have M., Tuithof M., Dorsselaer S., Kleinjan M., Penninx B.W.J.H., Batelaan N.M., Graaf R. (2020). Duration of anxiety disorder and its associated risk indicators: Results of a longitudinal study of the general population. Depress. Anxiety.

[9] Wang X., Lin J., Liu Q., Lv X., Wang G., Wei J., Zhu G., Chen Q., Tian H., Zhang K. (2022). Major depressive disorder comorbid with general anxiety disorder: Associations among neuroticism, adult stress, and the inflammatory index. J. Psychiatr. Res..

[10] Penninx B.W.J.H., Pine D.S., Holmes E.A., Reif A. (2021). Anxiety disorders. Lancet.

[11] Rabinowitz J., Williams J.B.W., Hefting N., Anderson A., Brown B., Fu D.J., Kadriu B., Kott A., Mahableshwarkar A., Sedway J. (2023). Consistency checks to improve measurement with the Hamilton Rating Scale for Anxiety (HAM-A). J. Affect. Disord..

[12] Chen C., Yu X., Belkacem A.N., Lu L., Li P., Zhang Z., Wang X., Tan W., Gao Q., Shin D. (2021). EEG-Based Anxious States Classification Using Affective BCI-Based Closed Neurofeedback System. J. Med. Biol. Eng..

[13] Thompson E. (2015). Hamilton Rating Scale for Anxiety (HAM-A). Occup. Med..

[14] Zorowitz S., Bennett D., Choe G., Niv Y. (2021). A recurring reproduction error in the administration of the Generalized Anxiety Disorder scale. Lancet Psychiatry.

[15] Müller-Putz G.R. (2020). Electroencephalography. Handbook of Clinical Neurology.

[16] Tasci G., Gun M.V., Keles T., Tasci B., Barua P.D., Tasci I., Dogan S., Baygin M., Palmer E.E., Tuncer T. (2023). QLBP: Dynamic patterns-based feature extraction functions for automatic detection of mental health and cognitive conditions using EEG signals. Chaos Solitons Fractals.

[17] Ancillon L., Elgendi M., Menon C. (2022). Machine Learning for Anxiety Detection Using Biosignals: A Review. Diagnostics.

[18] Shanok N.A., Jones N.A. (2024). EEG Asymmetry Characteristics in Relation to Childhood Anxiety Subtypes: A Dimensional Approach. Clin. Eeg Neurosci..

[19] Wang J., Fang J., Xu Y., Zhong H., Li J., Li H., Li G. (2022). Difference analysis of multidimensional electroencephalogram characteristics between young and old patients with generalized anxiety disorder. Front. Hum. Neurosci..

[20] Li Y., Qian L., Li G., Zhang Z. (2023). Frequency specificity of aberrant triple networks in major depressive disorder: A resting-state effective connectivity study. Front. Neurosci..

[21] Mokatren L.S., Ansari R., Cetin A.E., Leow A.D., Ajilore O.A., Klumpp H., Yarman Vural F.T. (2021). EEG Classification by Factoring in Sensor Spatial Configuration. IEEE Access.

[22] Park S.M., Jeong B., Oh D.Y., Choi C.-H., Jung H.Y., Lee J.-Y., Lee D., Choi J.-S. (2021). Identification of Major Psychiatric Disorders from Resting-State Electroencephalography Using a Machine Learning Approach. Front. Psychiatry.

[23] Liu W., Li G., Huang Z., Jiang W., Luo X., Xu X. (2023). Enhancing generalized anxiety disorder diagnosis precision: MSTCNN model utilizing high-frequency EEG signals. Front. Psychiatry.

[24] Qi X., Fang J., Sun Y., Xu W., Li G. (2023). Altered Functional Brain Network Structure between Patients with High and Low Generalized Anxiety Disorder. Diagnostics.

[25] Al-Ezzi A., Al-Shargabi A.A., Al-Shargie F., Zahary A.T. (2022). Complexity Analysis of EEG in Patients with Social Anxiety Disorder Using Fuzzy Entropy and Machine Learning Techniques. IEEE Access.

[26] Al-Ezzi A., Yahya N., Kamel N., Faye I., Alsaih K., Gunaseli E. (2021). Severity Assessment of Social Anxiety Disorder Using Deep Learning Models on Brain Effective Connectivity. IEEE Access.

[27] Mohammad F., Al-Ahmadi S. (2022). Human state anxiety classification framework using EEG signals in response to exposure therapy. PLoS ONE.

[28] Li Z., Wu X., Xu X., Wang H., Guo Z., Zhan Z., Yao L. (2022). The Recognition of Multiple Anxiety Levels Based on Electroencephalograph. IEEE Trans. Affect. Comput..

[29] Muhammad A.W., Basri A., Ghufran M., Taghreed S., Nada A., Nada A., Maryam A. (2020). Classification of Anxiety Disorders using Machine Learning Methods: A Literature Review. Insights Biomed. Res..

[30] Wang H., Liu X., Li J., Xu T., Bezerianos A., Sun Y., Wan F. (2020). Driving fatigue recognition with functional connectivity based on phase synchronization. IEEE Trans. Cogn. Dev. Syst..

[31] Ke G., Meng Q., Finley T., Wang T., Chen W., Ma W., Ye Q., Liu T.-Y. (2017). Lightgbm: A highly efficient gradient boosting decision tree. Adv. Neural Inf. Process. Syst..

[32] Chen T., Guestrin C. Xgboost: A scalable tree boosting system. Proceedings of the 22nd ACM Sigkdd International Conference on Knowledge Discovery and Data Mining.

[33] Prokhorenkova L., Gusev G., Vorobev A., Dorogush A.V., Gulin A. (2018). CatBoost: Unbiased boosting with categorical features. Adv. Neural Inf. Process. Syst..

[34] Sameen M.I., Pradhan B., Lee S. (2020). Application of convolutional neural networks featuring Bayesian optimization for landslide susceptibility assessment. Catena.

[35] Erickson N., Mueller J., Shirkov A., Zhang H., Larroy P., Li M., Smola A. (2020). Autogluon-tabular: Robust and accurate automl for structured data. arXiv.

[36] Koziarski M., Woźniak M. (2017). CCR: A combined cleaning and resampling algorithm for imbalanced data classification. Int. J. Appl. Math. Comput. Sci..

[37] Christou V., Miltiadous A., Tsoulos I., Karvounis E., Tzimourta K.D., Tsipouras M.G., Anastasopoulos N., Tzallas A.T., Giannakeas N. (2022). Evaluating the Window Size’s Role in Automatic EEG Epilepsy Detection. Sensors.

[38] He Y., Yang F. The effect of time window length on dynamic brain network analysis under various emotional conditions. Proceedings of the IEEE 6th Advanced Information Technology, Electronic and Automation Control Conference (IAEAC).

[39] Nunez P., Poza J., Gomez C., Barroso-Garcia V., Ruiz-Gomez S.J., Maturana-Candelas A., Tola-Arribas M.A., Cano M., Hornero R. Characterization of EEG Resting-state Activity in Alzheimer’s Disease by Means of Recurrence Plot Analyses. Proceedings of the 2019 41st Annual International Conference of the IEEE Engineering in Medicine and Biology Society (EMBC).

[40] Li J.W., Barma S., Mak P.U., Chen F., Li C., Li M.T., Vai M.I., Pun S.H. (2022). Single-Channel Selection for EEG-Based Emotion Recognition Using Brain Rhythm Sequencing. IEEE J. Biomed. Health Inform..

[41] Azinfar L., Rabbi A., Ravanfar M., Noghanian S., Fazel-Rezai R. Optimizing dynamical similarity index extraction window for seizure detection. Proceedings of the 2014 36th Annual International Conference of the IEEE Engineering in Medicine and Biology Society.

[42] Ouyang D., Yuan Y., Li G., Guo Z. (2022). The Effect of Time Window Length on EEG-Based Emotion Recognition. Sensors.

[43] Al-Ezzi A., Kamel N., Faye I., Gunaseli E. (2020). Review of EEG, ERP, and Brain Connectivity Estimators as Predictive Biomarkers of Social Anxiety Disorder. Front. Psychol..

[44] Li G., Zhong H., Wang J., Yang Y., Li H., Wang S., Sun Y., Qi X. (2023). Machine Learning Techniques Reveal Aberrated Multidimensional EEG Characteristics in Patients with Depression. Brain Sci..

[45] Cohen Z.D., DeRubeis R.J., Widiger T., Cannon T.D. (2018). Treatment Selection in Depression. Annual Review of Clinical Psychology.

[46] Massullo C., Carbone G.A., Farina B., Panno A., Capriotti C., Giacchini M., Machado S., Budde H., Murillo-Rodriguez E., Imperatori C. (2020). Dysregulated brain salience within a triple network model in high trait anxiety individuals: A pilot EEG functional connectivity study. Int. J. Psychophysiol..

[47] Newson J.J., Thiagarajan T.C. (2019). EEG Frequency Bands in Psychiatric Disorders: A Review of Resting State Studies. Front. Hum. Neurosci..

[48] Sviderskaia N.E., Prudnikov V.N., Antonov A.G. (2001). Characteristics of EEG signs of anxiety in human. Zhurnal Vyss. Nervn. Deiatelnosti Im. I P Pavlov..

[49] Buchsbaum M.S., Hazlett E., Sicotte N., Stein M., Wu J., Zetin M. (1985). Topographic EEG changes with benzodiazepine administration in generalized anxiety disorder. Biol. Psychiatry.

[50] Li G., Huang S., Xu W., Jiao W., Jiang Y., Gao Z., Zhang J. (2020). The impact of mental fatigue on brain activity: A comparative study both in resting state and task state using EEG. BMC Neurosci..

[51] Pfurtscheller G., da Silva F.H.L. (1999). Event-related EEG/MEG synchronization and desynchronization: Basic principles. Clin. Neurophysiol..

[52] Miskovic V., Ashbaugh A.R., Santesso D.L., McCabe R.E., Antony M.M., Schmidt L.A. (2010). Frontal brain oscillations and social anxiety: A cross-frequency spectral analysis during baseline and speech anticipation. Biol. Psychol..

[53] Engel A.K., Fries P., Singer W. (2001). Dynamic predictions: Oscillations and synchrony in top-down processing. Nat. Rev. Neurosci..

[54] Ochsner K.N., Silvers J.A., Buhle J.T. (2012). Functional imaging studies of emotion regulation: A synthetic review and evolving model of the cognitive control of emotion. Ann. N. Y. Acad. Sci..

[55] Yu X., Li Z., Zang Z., Liu Y. (2023). Real-Time EEG-Based Emotion Recognition. Sensors.

[56] Jang K.-I., Shim M., Lee S.M., Huh H.J., Huh S., Joo J.-Y., Lee S.-H., Chae J.-H. (2017). Increased beta power in the bereaved families of the Sewol ferry disaster: A paradoxical compensatory phenomenon? A two-channel electroencephalography study. Psychiatry Clin. Neurosci..

[57] Schoenberg P.L.A. (2020). Linear and Nonlinear EEG-Based Functional Networks in Anxiety Disorders. Anxiety Disord. Rethink. Underst. Recent Discov..

[58] Wang Y., Chai F., Zhang H., Liu X., Xie P., Zheng L., Yang L., Li L., Fang D. (2016). Cortical functional activity in patients with generalized anxiety disorder. BMC Psychiatry.

